# Effect of Preirradiation Fluoride Treatment on the Physical Properties of Dentin

**DOI:** 10.1155/2022/3215048

**Published:** 2022-03-17

**Authors:** Francis K. Mante, Aaron Kim, Kristi N. Truong, Kriti Mittal, Spoorthi Alapati, Sarah Hagan, Jie Deng

**Affiliations:** ^1^School of Dental Medicine, University of Pennsylvania, 240 South 40^th^ Street, Philadelphia, PA 19104, USA; ^2^School of Arts and Sciences, University of Pennsylvania, Philadelphia, PA 19104, USA; ^3^Perelman School of Medicine, University of Pennsylvania, Philadelphia, PA 19104, USA; ^4^School of Nursing, University of Pennsylvania, Philadelphia, PA 19104, USA

## Abstract

**Objective:**

To determine the effects of preirradiation fluoride treatments on the Knoop hardness of dentin.

**Materials and Methods:**

Human posterior teeth mounted into acrylic resin molds were polished with silicon carbide (SiC) abrasives and 3-micron diamond paste. The Knoop hardness of dentin was measured with a Leco hardness instrument. The teeth were divided into groups of ten teeth per group as follows: no treatment (control), treatment with silver diamine fluoride (SDF), MI varnish (MI), and cavity shield (CS). The teeth were exposed to 2 Gy of daily radiation for six weeks using an X-Rad 320ix biological irradiator. Hardness was measured weekly, before, during, and after irradiation. The teeth were stored in artificial saliva at 37^o^C between radiation treatments.

**Results:**

In preirradiation dentin, a Knoop hardness value of 58.8 (14.1) KHN was obtained. Treatment with SDF significantly increased KHN before irradiation. Immediately after radiation treatment, hardness was significantly reduced in all experimental groups. Postirradiation fluoride treatments increased the hardness of dentin to varying degrees.

**Conclusions:**

Preirradiation fluoride treatment does not provide protection from decreases in the hardness of dentin. Treatment of teeth with fluoride formulations after radiation progressively restores the hardness of dentin to different degrees.

## 1. Introduction

Radiation-related caries (RRC) is a common late effect of radiation therapy for head and neck cancer (HNC). Radiation causes destruction of salivary glands and the accompanying xerostomia has been identified as a major cause of RRCs [1]. Saliva plays an important role in maintaining oral/dental health by diluting sugars after intake of food and drink, buffering plaque acid and remineralizing enamel and dentin [2]. These effects of saliva provide a balance to the demineralization caused by biofilm acid. Saliva produced by radiation damaged glands has a lower volume, higher viscosity, and a lower pH [3] and does not effectively protect teeth against demineralization.

Although there were early reports of increased hardness and crystallinity in dentin irradiated during oral cancer treatment [4–6], more recent studies have provided details of direct detrimental effects of radiation on the physical properties of dentin and enamel. Exposure of dentin to irradiation during head and neck cancer treatment leads to demineralized and softened dentin regions [7–10]. This effect was demonstrated as low electron density images in demineralized dentin after radiation treatment [7]. Although damage was noticed both in the dentin and enamel regions, it was more severe in dentin. Using nanoindentation studies, Liang et al. (2015) found a significant dose-dependent decrease in hardness after 5 days of irradiation [8]. Subsequent decreases in hardness were progressive throughout the 6-week period of irradiation.

Deterioration of the physical properties of teeth has been attributed to postirradiation degradation of both dentin and enamel. Reduced crystallinity, disruption of hydroxyapatite crystals, disruption of protein-mineral interactions, and breakdown of protein content especially close to the DEJ have been reported in postirradiation dentin and enamel [11]. Consequently, it was suggested that the use of fluoride products may be beneficial for the prevention of radiation caries in patients' postirradiation.

These findings of direct effects of radiation on dentin demonstrate that the etiology of RRC should include a degradation of the physical properties of the tooth structure caused by irradiation. The degradation of the dentin substrate at the dentin-enamel junction explains the clinical reports of exfoliation of enamel with exposure of dentin in the cervical and labial aspects of incisors and the occlusal surfaces of molars in irradiated patients [1, 4, 10, 12].

Fluoride-based topical agents such as fluoride varnish and MI varnish (MI) have been investigated extensively as preventative agents for dental caries [13–18]. Cavity shield (CS, 3M ESPE, St. Paul, MN, USA) is a fluoride varnish used as a caries inhibitory agent [14]. The varnish reduces oral cariogenic bacteria and provides fluoride during the remineralization of tooth structure [15]. Remineralization in the presence of fluoride leads to the formation of fluorohydroxyapatite, which has a more dense crystal lattice and is more resistant to acid degradation than hydroxyapatite [16]. The American Dental Association (ADA) recommends that fluoride varnish should be applied 2–4 times per year in high risk patients [14, 15]. Fluoride varnish has been used to reduce the incidence of radiation caries [15–17]. For these patients, fluoride gels are applied daily in a custom tray.

MI varnish (GC America Alsip IL USA) is a 5% sodium fluoride varnish containing Recaldent, ™ which is a complex of casein phosphopeptides and amorphous calcium phosphate (CPP-ACP). Recaldent increases the level of calcium phosphate in dental plaque which would suppress the demineralization process and increase remineralization of dentin [18]. Clinical studies have shown that it is effective in correcting enamel white spot lesions after orthodontic treatment [19, 20]. However, the evidence for the effect of MI varnish on the remineralization of dentin is contradictory. Reynolds et al. [18] recommended that CPP-ACP should be incorporated into tooth paste for treatment of dentin hypersensitivity which would improve dentinal tubular occlusion and remineralize dentin. However, a laboratory study reported that the presence of CPP-ACP in a fluoride varnish did not significantly increase remineralization of dentin [21].

Silver diamine fluoride (SDF) has been used to arrest dental decay in several countries since 1970 [22]. In the U.S., SDF received the Food and Drug Administration (FDA) clearance in 2014 as a desensitizing agent for treating dental sensitivity. In 2016, it was recognized by the FDA with breakthrough therapy designation for caries treatment [23, 24].

The American Dental Association has developed guidelines for using SDF as a caries arresting medication [25], and a current dental terminology (CDT) code D1354 was approved in 2016 and revised in 2018. Clinical studies have found that SDF is more effective both for prevention and treatment of caries than fluoride varnish in both children and adult populations [26]. SDF fight carries by inhibition of biofilm growth, an antibacterial effect provided by silver and a high concentration of fluoride to remineralize dentin. It has been shown to be effective as a caries arrest, dentin desensitizer, and caries prevention agent [27]. It has also been reported that silver could exchange for small amounts of calcium to form silver-containing hydroxyapatite and promote an antibacterial effect [28].

Although topical fluoride is accepted world-wide for the prevention of caries before, during, and after radiation therapy, there is minimal information on the effect of pre-irradiation application of SDF on dentin on pre-venting the detrimental effects of irradiation.

The hypothesis of this research is that pretreatment of dentin with fluoride anticaries agents will prevent the degradation of hardness that accompanies oral cancer irradiation.

The purpose of the study was to determine the effects of pre and postirradiation SDF and other fluoride agents on the hardness of dentin and to determine whether fluoride delivery systems can prevent the deterioration of hardness of dentin. The study also observed the ability of teeth to remineralize after irradiation when treated with fluoride agents, SDF, MI, and CS.

## 2. Materials and Methods

Extracted premolar and molar teeth were collected from oral surgery practices in Philadelphia, USA. The teeth were collected from adult patients with no age restrictions and stored in 0.5 percent chloramine-T solution for up to 1 month before use. Since the teeth were not connected with the patient's identity at the time of collection, a waiver of informed consent was granted for the research by the University of Pennsylvania Institutional Review Board (IRB).

The teeth were cleaned of debris and soft tissue and mounted in acrylic resin molds. The teeth were ground with 120, 240, 400, 600, and 800 grit silicon carbide (SiC) abrasive papers and then polished with 3-micrometer diamond paste. The Knoop hardness of dentin was measured with a Leco Hardness Instrument M400-G1 (Leco Corp, St Josephs, MI, USA) under a load of 200 g at points 2 mm into the dentin at cusp tips. The teeth were divided into four different groups and treated as follows: no treatment (NT), treatment with 38% silver diamine fluoride (SDF: advantage arrest, elevate oralcCare, West Palm Beach, FL, USA), MI varnish (MI: GC America Inc., Alsip, IL, USA), and cavity shield (CS: 3M ESPE Dental Products, St. Paul, MN, USA). The fluoride agents used and their fluoride content are listed in Table 1.

The fluoride varnish was rubbed on the dentin surface using a cotton pellet for 1 minute. Excess material was left on the tooth surface and stored at 37^o^C with a 100% relative humidity chamber for 24 hours. SDF was applied for one minute and excess material was wiped off before storage in an incubator (Sanyo Scientific Wood Dale, IL, United States) at 37^o^ C and 100% relative humidity for 24 hours.

The samples received daily fluoride applications for 7 days. The teeth were then exposed to a daily dose of 2 Gy of radiation five days per week for six weeks using an X-Rad 320ix biological irradiator set to 320 KV, 12.5 mA.

Twenty Knoop hardness (KHN) measurements on ten teeth for each treatment condition were recorded before irradiation and once a week during the period of irradiation. The teeth were cleaned in a Branson 1510 ultrasonic cleaner before Knoop hardness measurements were made.

When the teeth were not being tested or treated, they were stored in artificial saliva (Pickering Laboratories, Mountain View, California, USA) at 37^o^C. The composition of artificial saliva used is listed in Table 2. After 6 weeks of irradiation, the teeth were treated with the fluoride delivery systems, and hardness was measured 1 week later. A second postirradiation fluoride treatment was performed, and hardness was measured 2 weeks after irradiation. The data obtained were analyzed using Kruskal–Wallis one-way ANOVA on ranks. Pairwise multiple comparisons were performed using Dunn's method.

## 3. Results

The effect of irradiation on KHN of fluoride-treated dentin is shown in Table 3. Knoop hardness values ranging from 55.0 (19.8) to 59.08 (15.7) was found for untreated dentin groups. These values were not significantly different from each other. After fluoride treatment and before radiation treatment, dentin treated with SDF had a significantly higher KHN than all other treatment groups. After 6 weeks of irradiation, the KHN of dentin was significantly lower for all control and experimental groups. Analysis of main effects show that treatment with SDF resulted in significantly higher KHN than other treatments before irradiation (*P* < 0.005). However, after 6 weeks of irradiation, all groups had KHN values significantly lower than preirradiation values, and these values were not significantly different from each other. Immediate postirradiation KHN values for all groups had values lower than those of the control untreated dentin. There was no significant difference between KHN of treatment groups (Table 3).

**Figure 1:**
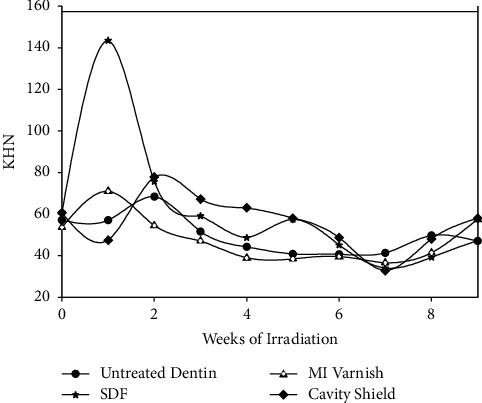
Dose/time dependence of KHN on irradiation.

Figure 1 shows the dependence of KHN on dosage and time of irradiation. Hardness at week one on the plot describes preirradiation values. After the first week of irradiation, there was a significant decrease in the KHN of SDF-treated dentin. During this same period, untreated dentin and cavity varnish-treated dentin exhibited a significant increase in hardness while MI-treated dentin showed no significant change in KHN. Subsequent irradiation led to progressive decreases in hardness for all groups, and a minimum hardness was reached at the sixth week of irradiation. All KHN values after six weeks of irradiation were lower than those of the nonirradiated controls for all groups, although the differences among them were not statistically significant (Table 3). Subsequent fluoride treatment led to gradually increasing KHN for all groups, although the rate of increase seemed to be more favorable for MI and CS treated dentin. The differences among the groups at nine weeks were not significantly different.

## 4. Discussion

The aim of this investigation was to test the hypothesis that pretreatment of dentin with anticaries fluoride agents can prevent the demineralization of teeth that accompanies high-energy X-ray irradiation, which is a common treatment modality for head and neck cancer. The results show that pretreatment of dentin with SDF results in a significant increase in KHN before irradiation. Other fluoride agents investigated did not significantly alter KHN. After 6 weeks of irradiation, the hardness of fluoride treated as well as control dentin was significantly lower than preirradiation values, and fluoride pretreatment did not prevent the decreases in hardness caused by irradiation (Table 3). We have used the surface hardness of dentin as a measure of the degree of mineralization or demineralization as several studies have reported that changes in the microhardness of dentin are directly related to its mineral content. [8–11, 29].

### 4.1. Hardness Changes after Fluoride Treatment

The results of this investigation show that treatment of dentin with SDF significantly increases KHN (Table 3). It has been reported that when applied to dentin, SDF forms a film with a high silver content, and silver-containing deposits are found on the surface and in dentin tubules to a penetration depth of 20–40 µm [30, 31]. This coating and penetration into dentin tubules is responsible for the desensitizing effect of SDF. Other investigators [27, 28, 31, 32] have demonstrated that SDF treatment leads to silver incorporation into hydroxyapatite and silver-containing HA compounds in dentin tubules could contribute to increased surface hardness of dentin.

The high fluoride content of SDF (44,800 ppm) [32] may also lead to a high rate of formation of fluorohydroxyapatite to contribute to an increase of surface hardness. The concentration of fluoride in SDF is important because lower concentrations of fluoride, 12% (14,000 ppm) and 30% SDF (35,400 ppm), are not as effective in arresting caries as 38% SDF solutions [27, 33, 34]. The significant increase in dentin hardness explains the ability of SDF-treated teeth to resist demineralization and halt the caries process and to simultaneously prevent the formation of new caries. This makes SDF more effective than other caries preventive agents, with lower fluoride concentrations such as stannous fluoride (1,100 ppm) and 5% sodium fluoride (22600 ppm) gels in preirradiated patients. However, application of SDF to carious tooth structure leads to the formation of a black stain which may limit its application to posterior teeth.

Preirradiation treatment of dentin with CS and MI did not significantly increase the hardness of the dentin surface. Although several studies have demonstrated that the application of fluoride *in vivo* leads to remineralization of demineralized dentin [35–37], the effects of ex vivo application of fluoride varnishes on intact dentin hardness are equivocal and may depend on acidity, fluoride concentration, and carrier (other constituents) of the varnish [38, 39]. Investigations of the protective effects of fluoride gels have shown that they are more effective against enamel caries when flouride is present in the oral fluids around the tooth during an acid challenge. It is therefore not surprising to find differences in the in vivo and ex vivo responses of dentin on exposure to fluoride varnishes. In vivo topical fluoride application to enamel and dentin is apparently accompanied by the formation of new crystals from available calcium that incorporate fluoride. The new crystals, fluoroapatite, are larger and impart higher density, radio-opacity, and greater resistance to acid breakdown [40]. In the absence of calcium ions, ex vivo application does not yield an appreciable increase in mineralization unless much higher concentrations of fluoride are present, as shown by SDF application in this study. Although the artificial saliva used contained calcium ions, the concentrations did not appear to be enough to effect a significant change in hardness.

MI is a 5% sodium fluoride varnish containing Recaldent™, which is a complex of casein phosphopeptides and amorphous calcium phosphate (CPP-ACP). Recaldent increases the level of calcium phosphate in dental plaque which would depress the demineralization process and raise the remineralization process [18]. The data appear to show that in the absence of a demineralization challenge, the enhancement of calcium and phosphate provided by MI does not significantly change the hardness of dentin. There are contradictory reports of the effect of MI on caries prevention in the scientific literature. In some studies [19, 20], a significant reduction of caries depth and white spot lesions around orthodontic brackets were attributed to the presence of Ca^++^ and PO_4−_. Others report that calcium and phosphate compositions in dentifrice do not seem to enhance or inhibit the performance of fluoride varnishes [21, 41–43]. In general, the higher the content of fluoride in a dentifrice, the better its ability to prevent caries. A study that compared the ability of two fluoridated dentifrices, one containing 5,000 ppm (PreviDent 5000 Plus®) and the other 1,100 ppm (Winterfresh Gel®), to reverse primary root caries lesions concluded that the 5,000 ppm fluoride dentifrice was significantly better at preventing the lesions than the 1,100 ppm fluoride dentifrice [44]. It is postulated that the anticariogenic mechanism of CPP-ACP is the localization of amorphous calcium phosphate at the tooth surface, where it buffers free calcium and phosphate ion activity during an acid challenge and maintains a state of supersaturation of calcium and phosphate ions on the enamel surface [45]. This process results in a decrease in demineralization during a cariogenic challenge and an increase in the subsequent remineralization of the enamel [18, 46, 47]. Dentin samples in this study were not exposed to any demineralizing challenge before irradiation and did not show a significant change in hardness after treatment with MI. It also appears that the demineralization of dentin caused by radiation is more pervasive than acid demineralization. As reported [11, 48], radiation damage of dentin includes a breakdown of protein content which can lead to a disruption of protein-mineral interactions.

### 4.2. Hardness Changes after Irradiation

The increases in surface hardness obtained on SDF-treated dentin were reversed rapidly after radiation treatment was begun. The KHN decreased progressively to a minimum value at the 6th week of irradiation. The effect of irradiation on the hardness of SDF-treated dentin may be explained by the interactions of x-rays with metallic components of the fluoride agent [49–51].

When teeth treated with fluoride agents are exposed to x-rays, interactions with the metallic components will lead to the discharge of photons by the photoelectric and Compton effects. In the photoelectric effect, an X-ray photon uses up its energy to eject an electron from an atom. This leaves the atom in an ionized (i.e., charged) state. The ionized atom then returns to the neutral state with the emission of an X-ray characteristic of the atom. The second major effect is Compton (incoherent) scatter, where the X-ray photon hits an atom and ionizes an electron but does not use up all its energy. The photon then scatters in a different direction with a bit less energy, and the free electron goes about doing damage. Electrons ejected from the metals can act as secondary sources of X-rays. Each time a photon ejects an electron–ionizes an atom–the probability of photoelectric absorption is approximately proportional to (Z/*E*)^3^, where *Z* is the atomic number of the tissue atom and *E* is the photon energy [49]. Thus, higher atomic weight metals may increase the energy (dose) kerma (kinetic energy released in matter) that dentin absorbs from photon interactions. While the mechanism may operate in the presence of all fluoride agents, the damage is more dramatic when radiation is applied to SDF-treated teeth due to the presence of silver with a relatively high atomic number of 47. The metallic components in the other fluoride agents used are sodium (atomic number of 11) and calcium (atomic number of 20) which would be expected to show a much less multiplying effect of radiation. Correspondingly, the decrease in hardness after initial irradiation is strongest for dentin treated with SDF and lowest for sodium fluoride varnish.

### 4.3. Structural Effects of Irradiation

The structural changes in irradiated dentin that lead to decreases in hardness can be explained by the decarboxylation of the tissue. In normal dentin, calcium from apatite crystals is bound electrostatically to the carboxylate and phosphate side chains of collagen. These linkages reinforce dentin by intra and extra fibrillar mineral deposits which control its elastic behavior [52]. When exposed to high X-ray energy, the decoupling of calcium from collagen side chains leads to decarboxylation [8, 13]. The degradation of dentin can be observed as a decrease in both the crystallinity [13] and the protein-to-mineral ratio in dentin [53]. The loss of mineral-organic linkages leads to a degradation of mechanical properties and can induce microcracks in dentin [13]. Remineralization occurs by the formation of new crystals in demineralized zones [54]. If fluoride ions are present, they are incorporated into the new crystals to yield fluorapatite which is more resistant to demineralization than hydroxyapatite [53, 54].

The KHN of control dentin and cavity varnish-treated dentin both rose significantly after one week of irradiation. Based on previous reports [13], we attribute this increase in KHN to the loss of bound water which may not be easily replaced during storage in artificial saliva. Subsequent irradiation resulted in progressive decreases in the hardness of dentin which may be attributed to interaction of radiation with water, degradation of the organic content of dentin, decreased crystallinity of hydroxyapatite crystals, and a disruption of the interaction between inorganic and organic components of dentin [10–13]. KHN of dentin treated with MI showed a slight increase in hardness when compared to control dentin. However, subsequent irradiation led to a similar pattern of hardness decreases found for all groups.

Fluoride treatment after irradiation resulted in a gradual restoration of hardness for all treatment groups. Although the KHN values after irradiation appear to increase at a faster rate for MI and CS treated dentin, the values for all experimental groups after two weeks of postirradiation fluoride treatment were not significantly different.

### 4.4. Limitations of the Study

This in vitro study is subject to limitations that should be considered in the interpretation of the results. Extracted teeth were collected from several patients, and this variability has resulted in high standard deviations in our data. Another reason for variability in the data is that dentin is anisotropic [13, 55], and hardness measurements are subject to differences in orientation of dentin tubules. To minimize variability in the data, measurements were made on surfaces that were flat and parallel to the instrument table. Measurements were limited to cuspal dentin areas. Also, isolated teeth do not perfectly represent teeth in the oral cavity as fluoride and radiation effects ion of teeth will affect the exposure to radiation. Consequently, the dosage of radiation may not accurately represent that received by teeth in patients undergoing radiation treatment. Furthermore, the research would have benefited from a prolonged period of fluoride treatments and data collection after irradiation to determine if the different fluoride treatments confer long-term differences on the recovery of dentin hardness after irradiation.

Due to damage to salivary glands in patients who receive radiation for head and neck cancer, the composition of their saliva is expected to be altered in relation to pH. Artificial saliva used in the study may provide protection to teeth that are already present in patients. We therefore recommend that further studies are needed to fully elucidate the effect of fluoride treatments to promote recovery of physical properties of the teeth after oral cancer radiation treatment.

## 5. Conclusions

The following conclusions have been made within the constraints of the study:

1. Treatment of dentin with SDF results in a significant increase in hardness
2. Radiation treatment lowers the hardness of dentin
3. Fluoride treatments including SDF do not prevent the degradation of the hardness of dentin due to irradiation
4. Fluoride treatment after irradiation gradually increases the hardness of dentin

## Figures and Tables

**Table 1:** Fluoride agents used for the study.

Fluoride agents used for the study			
Material	Manufacturer	Fluoride content (%)	Batch #
Silver diamine fluoride	Elevate Oral Care (West Palm Beach, FL, USA)	38	17228A
MI varnish	GC America Alsip (IL, USA)	5	1710191
Cavity shield	3M ESPE (St. Paul, MN, USA)	5	A38664

**Table 2:** Composition of artificial saliva.

Composition of artificial saliva	
Sodium chloride	0.4 g/L
Sodium phosphate monobasic dihydrate	0.69 g/L
Potassium chloride	0.4 g/L
Calcium chloride dihydrate	0.906 g/L
Urea	1.0 g/L
Sodium sulfide nonahydrate	0.005 g/L
Proclean	0.3 mL/L
pH adjusted to 7 with HCl and NaOH	

**Table 3:** Effect of irradiation on KHN of dentin.

Treatment	KHN of polished dentin	KHN after fluoride treatment	KHN immediate postirradiation	KHN 9 weeks postirradiation
Control (no fluoride treatment)	58.8(14.1)^b^	—	39.0 (13.3) ^ce^	47.0 (15.9) ^bde^
SDF	55.7 (22.1)^b^	136.6 (13.7)^a^	36.6 (11.3) ^cf^	47.2 (16.9) ^bdf^
MI	55.0 (19.8)^b^	66.2 (11.3) ^bg^	37.2 (9.3)^c^	57.4 (13.9) ^bdg^
CS	59.08(15.7)^b^	48.5 (27.5) ^bh^	33.5 (7.1)^c^	58.0 (15.8) ^bdh^

Note: mean values with the same superscript are not significantly different (*P* < 0.05).

## Data Availability

The Knoop Hardness (KHN) data used to support the findings of this study are available from the corresponding author upon request.
